# The role of SIRT1 in the process of *Toxoplasma gondii* infection of RAW 264.7 macrophages

**DOI:** 10.3389/fmicb.2022.1017696

**Published:** 2022-11-17

**Authors:** Kai Dong, Ziyang Jiang, Jianhui Zhang, Hanxiao Qin, Jianping Chen, Qiwei Chen

**Affiliations:** ^1^Department of Parasitology, West China School of Basic Medical Sciences and Forensic Medicine, Sichuan University, Chengdu, China; ^2^Animal Disease Prevention and Food Safety Key Laboratory of Sichuan Province, Chengdu, China

**Keywords:** *Toxoplasma gondii*, macrophages, SIRT1, innate immunity, autophagy

## Abstract

*Toxoplasma gondii* is an opportunistic pathogenic protozoan that can infect almost all kinds of warm-blooded animals, including humans. *T. gondii* can evade the host's immune response, a process known as immune evasion. Our main objective was to evaluate the role played by Sirtuin1 (SIRT1) [one of the sirtuins (SIRTs) that are a family of nicotinamide adenine dinucleotide (NAD)-dependent histone deacetylases (HDACs)] in the *T. gondii* infection of RAW264.7 macrophages. In this study, we evaluated and observed alterations in the activity, expression, and localization of SIRT1 and assessed its involvement in the CD154/IFN-γ (CD40 ligand/interferon gamma) killing pathway and in autophagy during *T. gondii* infection. The inhibition of SIRT1 in host cells effectively reduced the number of intracellular tachyzoites, and the mechanism behind this effect might be the upregulation of IRGM1 [murine ortholog of IRGM (immunity-related GTPase family M)] and the initiation of autophagy. To the best of our knowledge, our study is the first to prove that *T. gondii* infection upregulates SIRT1 in RAW264.7 cells and that the inhibition of SIRT1 reduces the number of intracellular tachyzoites. Moreover, the upregulation of IRGM1 and the activation of autophagy may contribute to the intracellular inhibition of *T. gondii* caused by SIRT1 inhibition.

## Introduction

*Toxoplasma gondii* is an opportunistic pathogenic protozoan that is capable of infecting almost all kinds of warm-blooded animals, including humans (Dubey, 2009). *T. gondii* infection can be divided into congenital and acquired infections. Although latent infection occurs commonly in the healthy population, immunocompromised individuals (such as AIDS patients) are prone to death after infection due to severe complications such as Toxoplasma encephalopathy. *T. gondii* infection during pregnancy may lead to abortion, stillbirth, brain injury, deformity, eye injury, etc. (Torgerson and Mastroiacovo, 2013; Torgerson et al., 2015). *T. gondii* is capable of immune evasion, which is crucial to its survival and proliferation within the host (Lima and Lodoen, 2019). However, existing studies on the interaction between *T. gondii* and the host fail to fully reveal its immune evasion mechanism. Therefore, an urgent evaluation of the underlying mechanisms responsible for its immune evasion is necessary to develop effective preventive and therapeutic modalities.

SIRT1 is currently one of the most widely studied sirtuins (NAD^+^-dependent histone deacetylases). It is involved in a variety of physiological regulatory mechanisms including fatty acid oxidation, stress tolerance, insulin secretion, and glucose synthesis (Baur et al., 2012; Vachharajani et al., 2016; Jeśko et al., 2017), mostly *via* the regulation of energy metabolism. Therefore, a close connection between SIRT1 and autophagy has been widely reported (Lapierre et al., 2015). During infection, *T. gondii* utilizes nutrients of the host cell to metabolize and proliferate, which may lead to changes in the state of intracellular energy metabolism, thereby affecting important molecules or signal transmission related to energy metabolism. Other than the abovementioned points, our previous study showed that stimulation with resveratrol (a SIRT1 regulator) resulted in the high expression of the microtubule-associated protein 1A/1B-light chain 3 (LC3 type II) protein in host cells during *T. gondii* infection, and significantly reduced the number of intracellular tachyzoites (Chen et al., 2019). Therefore, SIRT1 may play an important role in the invasion and proliferation of *T. gondii* and also the elimination of intracellular tachyzoites by the host. In mice and humans, the control of acute *T. gondii* infection was reported to depend on IFN-γ and related pathways. IFN-γ*-*induced immune-related GTPases (IRGs) have been shown to destroy tachyzoites-containing parasitophorous vacuoles with some autophagy-related proteins in mice and humans (Howard et al., 2011). The entire LC3-binding system of ATG5 (autophagy-related 5), ATG7 (autophagy-related 7), ATG3 (autophagy-related 3), and the ATG5–ATG12–ATG16L1 (autophagy-related 5–autophagy-related 12–autophagy-related 16-like 1) complex is involved in the IFN-γ-dependent recruitment of anti-*T. gondii* IRGs (Choi et al., 2014; Haldar et al., 2014; Ohshima et al., 2014). In mice particularly, IFN-γ confers some protection against *T. gondii* infection by upregulating IRGM1 [murine ortholog of IRGM (immunity-related GTPase family M)]. The latter is a human protein that has been recently highlighted for its contribution to autophagy upon infections, the murine ortholog of IRGM is called IRGM1 levels, but the exact mechanism remains unclear (Howard et al., 2011). At the same time, CD40 was reported to resist *T. gondii* infection by activating the classical autophagy pathway (Portillo et al., 2010). In this study, we aimed to explore the role of SIRT1 in these two pathways against *T. gondii* infection.

In conclusion, this study focused on the role of SIRT1 in the invasion of the RH strain of *T. gondii* and the clearance of parasites by host immune cells to demonstrate the mechanism of the immune evasion of *T. gondii* and lay the foundation for further exploration of the potential of SIRT1 for its application as an anti-*T. gondii* therapeutic target.

## Materials and methods

### Experimental materials

A murine macrophage-stable cell line (RAW 264.7) and a human foreskin fibroblast (HFF) cell line were purchased from Jennio Biotech Co., Ltd., Guangzhou, China.

The type I virulent RH strain of *T. gondii* (generously donated by Guizhou Medical University, China) was stored in a phosphate-buffered saline (PBS) solution containing 20% glycerin in liquid nitrogen.

### Major experimental reagents and equipment

The following reagents and equipments were used in the studies: SRT1720 (hydrochloride) (CAS No.: 1001645, MCE, USA), EX527 (CAS No.: 49843-98-3, MCE, USA), Rapamycin (CAS No.: 53123-88-9, MCE, USA), recombinant Interferon-γ (Cat. No.: I4777, Sigma-Aldrich, USA), recombinant mouse TRAP/CD40L protein (Active) (ab220551, Abcam, USA), anti-SIRT1 antibody [SirT1 (1F3) Mouse mAb #8469, Cell Signaling Technology, USA], anti-LC3 antibody [LC3A/B (D3U4C) XP Rabbit mAb #12741, Cell Signaling Technology, USA], anti-mTOR antibody [mTOR (7C10) Rabbit mAb #2983, Cell Signaling Technology, USA], anti-Phospho-mTOR antibody [Phospho-mTOR (Ser2448) (D9C2) XP Rabbit mAb #5536, Cell Signaling Technology, USA], anti-IRGM1 antibody [IRGM (E6P7W) Rabbit mAb #71950, Cell Signaling Technology, USA], anti-beta-Actin mouse monoclonal antibody (Cat. No.: T0022, Affinity, China), LysoTracker™ Red DND-99–Special Packaging (Cat. No.: L7528, Thermo Fisher, USA), ProLong Gold Antifade Mountant with DAPI (Cat. No.: P36935, Thermo Fisher, USA), Goat anti-Mouse Alexa Fluor 488 (Cat. No.: A-11017, Thermo Fisher, USA), microplate reader (Sunrise, TECAN, Switzerland), Confocal Microscope (LSM 710, Zeiss, Germany), and Transmission Electron Microscope (TEM) (HT7800, Hitachi, Japan).

### Experimental methods

#### Cell cultivation and parasite culture

A murine macrophage-stable cell line (RAW 264.7) was cultured at 37°C in a 5% CO_2_-95% air mixture in a RPMI 1640 (Roswell Park Memorial Institute Medium 1640), supplemented with 10% fetal bovine serum (FBS) and 100 μg/ml of antibiotics (penicillin and streptomycin; Ameresco, USA), with passage every 3 days. When the cells were over 80% confluent, 5 ml of trypsin with 0.25% ethylenediaminetetraacetic acid (EDTA) and phenol red was introduced to digest the cells before they were washed off with fresh medium by pipetting for further seeding procedures.

The *T. gondii* RH strain was removed from liquid nitrogen, allowed to recover in a water bath at 37°C for 30 min, and then maintained *in vitro* by serial passage in human foreskin fibroblasts (HFFs). When over 90% of the infected cells were lysed, the parasites were obtained together with the attached cells with a gentle scraping by a cell scraper, followed by centrifugation at 100 *g* for 10 min at 4°C before the supernatants were collected. Next, the mixture was passed through a 25-gauge syringe needle several times, centrifuged at 2,000 *g* for 10 min to remove the supernatants, and resuspended in either PBS (for infection) or a RPMI 1640 medium supplemented with 5% FBS (Endo buffer) at 37°C in 5% CO_2_ (for treatment). The concentration of *T. gondii* tachyzoites (Unit: cells /ml) was calculated by a hemocytometer under an optical microscope.

#### Intracellular *Toxoplasma gondii* growth

RAW264.7 cells were collected and loaded in a 24-well cell culture plate (The cell slides were inserted in advance) at 1.5 × 10^5^ cells/per well and infected with RH tachyzoites at a ratio of 5:1 (tachyzoites:cells), followed by stimulation of SRT1720 (a SIRT1 stimulator), EX527 (a SIRT1 inhibitor), IFN-γ or CD154 (as known as CD40L, a ligand of CD40) at different time points. Intracellular *T. gondii* growth was performed after stimulation by a standard Wright's staining exclusion test to determine the number of intracellular RH tachyzoites. Subsequently, 200 infected cells were randomly selected on each cell slide, the number of intracellular *T. gondii* was counted, and the number of *T. gondii* in each parasitophorous vacuole was recorded. Next, a scatter plot was drawn, the mean number (X ± S) was calculated, and a statistical analysis was carried out.

#### NAD^+^/NADH ratio assay

In the NAD^+^/NADH ratio assay, NAD^+^ and NADH levels were measured using NAD^+^/NADH assay kits, with WST 8 (water-soluble tetrazolium salt-8) according to the manufacturer's instructions (Beyotime, China). Ethanol was oxidized to acetaldehyde by alcohol dehydrogenase (ADH). During this reaction, NAD^+^ was reduced to NADH; the generated NADH in the electron coupling reagent 1-mPMS (1-methoxy-5-methylphenazinium methyl sulfate) reduced WST-8 to orange-yellow formazan with a maximum absorption peak of approximately 450 nm. The amount of NADH was measured as follows: After heating in a water bath at 60°C for 30 min, the NAD^+^ present in the sample decomposed and only NADH remained. NADH reduced WST-8 to formazan, and the amount of formazan produced by the reaction was determined by colorimetry. Finally, the amount of NADH in the sample was determined. A microplate reader (Sunrise, TECAN, Switzerland) was employed to measure the absorbance at a wavelength of 450 nm. Determination of NAD^+^ and the NAD^+^/NADH ratio: Based on the total amount of NAD^+^ and NADH and the amount of NADH obtained in the previous two steps, the number of NAD^+^ molecules in the sample and the NAD^+^/NADH ratio were calculated using the following formula:

[NAD^+^] = [NAD_total_]-[NADH]

[NAD^+^]/[NADH] = ([NAD_total_]-[NADH])/[[NADH]]

#### Detection and analysis of protein production

The macrophages (2.5 × 10^6^) in each well of a 12-well tissue culture plate were lysed with a Western-PI (propidium iodide) lysis buffer supplemented with phenylmethylsulfonyl fluoride, followed by detection of the amount of total protein in each sample. Western blot analysis of the prepared protein samples was performed following standard protocols. Approximately 20–30 μg of protein was loaded in each lane of a 12% acrylamide gel. Proteins were separated at 120 V until the dye front reached the bottom of the gel, followed by transferring the gels to polyvinylidene fluoride (PVDF) membranes (Whatman) in transfer buffer [25 mM Tris–HCl, 192 mM glycine, 20% methanol, and 0.02% sodium dodecyl sulfate (SDS), pH 8.3], where they were held at 200 mA for 60 min. The membranes were then soaked in a blocking solution consisting of a PBS–Tween 20 buffer (1 × PBS, 0.1% Tween 20) supplemented with 1% bovine serum albumin (BSA; Sigma) for 2 h, before incubating overnight at 4°C individually with an anti-SIRT1 antibody and an anti-beta-Actin antibody at the corresponding dilutions. All primary antibodies were used at a 1:1,000 dilution, and horseradish peroxidase (HRP)-conjugated anti-rabbit/anti-mouse immunoglobulin secondary antibodies (Affinity, China) were used at a 1:2,500 dilution. The membranes were then visualized with an enhanced chemiluminescence Western blotting detection kit (Beyotime, China).

#### Intracellular SIRT1 and lysosome localization

SIRT1 in RAW 264.7 mouse macrophage cells was labeled and visualized with mouse anti-SIRT1 and Alexa Fluor 488 goat anti-mouse immunoglobulin G (IgG) (green) antibodies (Thermo Fisher, USA), respectively. Lysosomes were visualized using the LysoTracker Deep Red (Thermo Fisher, L7528, USA). Nuclear DNA was stained with the blue fluorescent DAPI (Thermo Fisher, Prolong Gold Antifade reagent with DAPI, USA). SIRT1 localization within the cell was observed using a confocal microscope (LSM 710, Zeiss, Germany).

#### Observation of intracellular tachyzoites by transmission electron microscopy

Macrophages (4 × 10^6^) in each well of a 6-well tissue culture plate were scraped and collected in a centrifuge tube and centrifuged at 1,500 rpm (revolutions per minute) for 10 min. The supernatant was discarded, 0.5% glutaraldehyde was slowly added along the tube wall, and the cells were resuspended at 4°C. The samples were centrifuged again at 12,000 rpm for 15 min. After being fully rinsed in distilled water, the samples were dehydrated in graded acetone series and embedded in SPI-Pon812. Ultrathin sections were cut with a Leica EM UC7 ultramicrotome, attached to copper grids with a Formvar film, then stained with 2% uranyl acetate and Reynolds lead citrate, and examined under a Hitachi HT7800 electron microscope at 80 kV.

### Statistics

The data were analyzed using the Student's *t*-test for the comparison of two groups or by a one-way analysis of variance (ANOVA) for the comparison of three or more groups using GraphPad Prism (version 5) software. *P* < 0.05 were considered significant.

## Results

### Changes of SIRT1 in *Toxoplasma gondii*-infected murine macrophages at different stages

**Figure 1:**
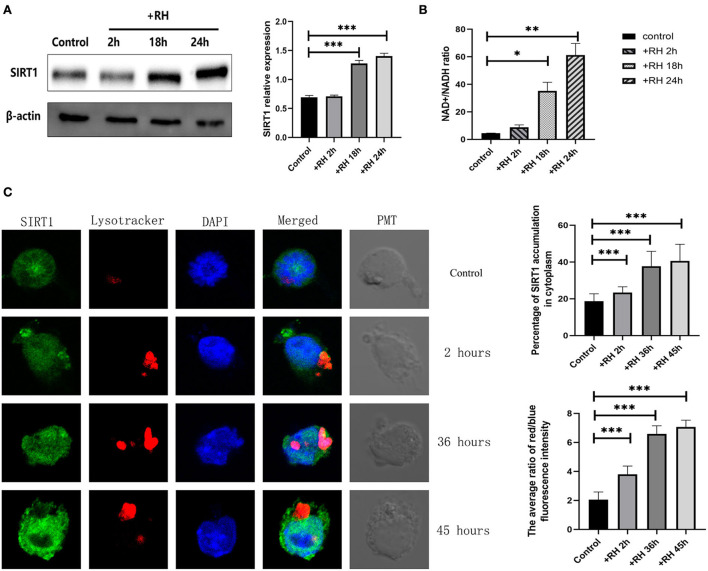
Changes in the expression, activity, location, and degradation of Sirtuin 1 (SIRT1) at different stages of *Toxoplasma gondii* infection. RAW 264.7 macrophages were either infected with RH tachyzoites at a ratio of 5:1 (parasite/host cell) for 2 h or left in the culture plate without infection. The NAD^+^/NADH (nicotinamide adenine dinucleotide) samples of the macrophages were extracted at 2, 18, and 24 h after infection of uninfected cells. The specific details of NAD^+^/NADH ratio measurement are illustrated in the NAD^+^/NADH ratio assay of the Experimental Methods section. The total protein of the host cells was extracted at 2, 18, and 24 h after infection, and the total SIRT1 level was detected by Western Blot. The confocal samples of the macrophages were extracted at 2, 36, and 45 h after infection of uninfected cells. **(A)** *T. gondii* infection leads to an increment in the NAD^+^/NADH ratio of host cells over time, indicating the upregulation of SIRT1 activity. The ratio of NAD^+^/NADH in murine macrophages did not increase significantly at 2 h of RH tachyzoite infection, but at 18 and 24 h, the ratio of NAD^+^/NADH increased significantly and increased with the prolongation of infection time. **(B)** *T. gondii* infection upregulated SIRT1 levels in host cells over time. **(C)** Fluorescent graphics showing the SIRT1 expression and lysosome activity of macrophages infected with RH tachyzoites for 2, 36, and 45 h. In the absence of *T. gondii*, SIRT1 in murine macrophages is predominantly expressed in the nucleus. With the prolongation of RH tachyzoite infection time, the expression of SIRT1 in host cells increased. Furthermore, the distribution of SIRT1 in murine macrophages also gradually shifted mainly from the nucleus to the cytoplasm, and gradually accumulated in the location of intracellular *T. gondii*. It also resulted in an upregulated activity of lysosomes, as seen by the red fluorescence. The intracellular *T. gondii* gradually accumulated in the lysosomes. These results were obtained from three independent experiments. The values shown are the means ± SEM from three independent experiments. SIRT1, green fluorescence; Lysotracker, red fluorescence; DAPI, blue fluorescence, PMT (photomultiplier tube), the light field with PMT as the detector and laser 488 nm as the light source. *P < 0.05, **P < 0.01, and ***P < 0.001.

To clarify the role of SIRT1 in T. gondii infection, the NAD+/NADH ratio, alterations of SIRT1 in expression, and subcellular localization and presence of lysosomes were examined by the NAD+/NADH ratio assay, Western blot, and confocal microscopy (Figure 1). We examined the indices mentioned above by directly adding RH tachyzoites into RAW264.7 cells for different time points. SIRT1 is one of the Sirtuins that are conserved proteins and a family of nicotinamide adenine dinucleotide (NAD)-dependent histone deacetylases (HDACs). SIRT1 activity is regulated by the NAD^+^ availability. In fact, the NAD^+^/NADH ratio upregulation in the cell promotes the SIRT1 protein deacetylase activity. Therefore, we chose to measure SIRT1 activity by measuring this value (Fang et al., 2021). *T. gondii* infection has been shown to upregulate the NAD^+^/NADH ratio in host cells (Figure 1B). The results revealed that *T. gondii* increased the host's SIRT1 expression level over time in macrophages (Figure 1A). Given that SIRT1 is degraded by lysosomes during cellular senescence (Wang et al., 2021), we introduced LysoTracker to label lysosomes and tested the changes in lysosomes of host cells in the presence of *T. gondii* infection under fluorescence microscopy. The results revealed that the infection led to an increased translocation of SIRT1 in the host cell cytoplasm from the nucleus as well as an upregulated activity of lysosome (Figure 1C). However, we did not detect the degradation of SIRT1 by lysosomes in host cells in the presence of *T. gondii* infection (Figure 1C).

### The impact of SIRT1 on the number of intracellular tachyzoites

**Figure 2:**
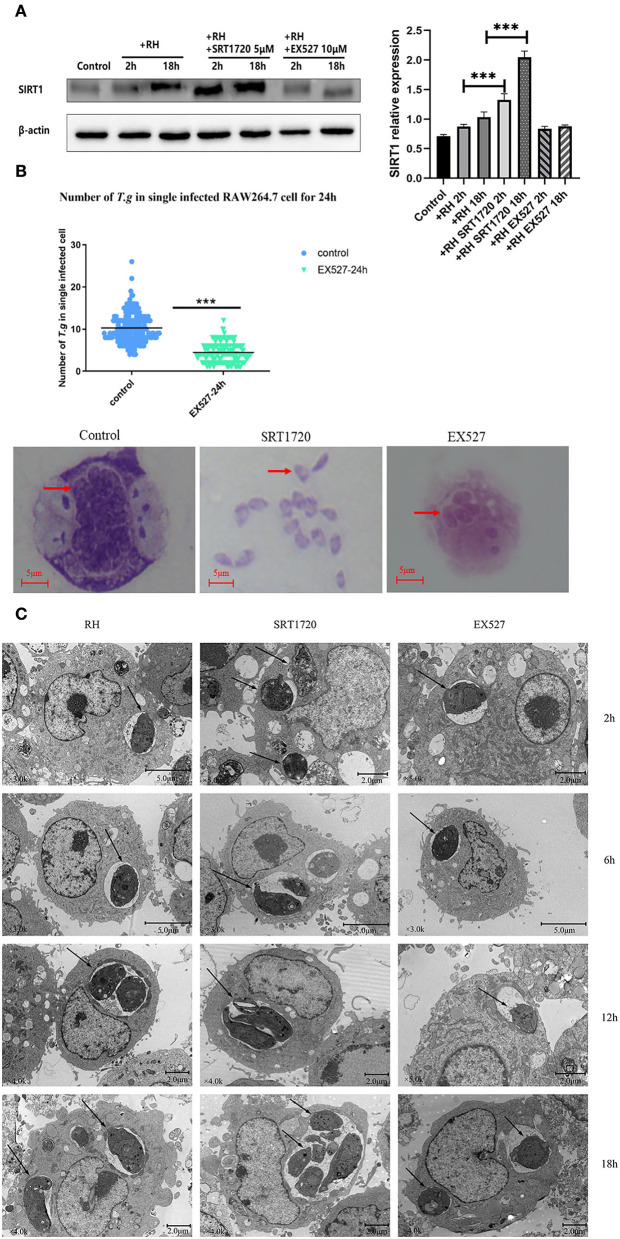
Intracellular growth of RH tachyzoites with SRT1720 and EX527 *in vitro*. RH tachyzoites were harvested to infect RAW 264.7 mouse macrophages at an infection ratio of 5:1 (parasite/host cell) for 2 h, followed by the removal of excess parasites and stimulation with SRT1720 (5 μM) and EX527 (10 μM) for 24 h and further examination by staining with Wright's stain. RAW 264.7 cells stimulated with SRT1720 (5 μM) and EX527 (10 μM) for 2, 6, 12, or 18 h were collected and further examined by transmission electron microscopy (TEM) or lysed for Western Blot. We adjusted the protein concentration of each group of samples to be consistent and then carried out the experiment. Anti-SIRT1 antibody and anti-beta-Actin antibody were used at a 1:1,000 dilution. **(A)** *T. gondii* infection upregulated SIRT1 levels in host cells over time. Infected macrophages treated with SRT1720 exhibited an upregulation of SIRT1 at 2 and 18 h compared to that for the *T. gondii* infection group. Infected macrophages treated with EX527 showed a downregulation of SIRT1 at 2 and 18 h, compared to that for the *T. gondii* infection group. **(B)** A scattered distribution chart reveals the mean intracellular tachyzoite counts. Values represent actual numbers (scattered dots) and means (black lines) from three independent experiments; in each experiment, at least 200 infected cells were counted. The average number of *T. gondii* tachyzoites in macrophages stimulated by EX527 was significantly reduced compared to the control group. The average number of tachyzoites in the control group was about 10, while the average number of tachyzoites in the EX527 group dropped to about 5. T.g, *T. gondii* tachyzoites ***P < 0.001. The infected macrophages treated with SRT1720 had a much heavier intracellular tachyzoite burden than those incubated with EX527. **(C)** The number of *T. gondii* tachyzoites in the cell increased with the duration of the infection. At the same time, compared with the control group, the group with SRT1720 addition had more intracellular *T. gondii* tachyzoites. In contrast, the EX527 stimulation group had fewer intracellular *T. gondii* tachyzoites than the control group. These results were obtained from three independent experiments. The values shown are the means ± SEM from three independent experiments. RH, *T. gondii* RH strain. ***P < 0.001.

Based on the above experiments, *T. gondii* was likely to affect the host's SIRT1 for survival and proliferation. Therefore, EX527 (a SIRT1 inhibitor) and SRT1720 (A SIRT1 stimulator) were employed to interfere with the activity of host cell SIRT1 to figure out whether the alteration of SIRT1 could have an impact on the intracellular parasite load. In addition to its known effects on SIRT1 activity, SRT1720 upregulated the SIRT1 expression level in *T. gondii*-infected macrophages, while EX527 downregulated the SIRT1 expression level in *T. gondii*-infected macrophages (Figure 2A). We found that the number of intracellular tachyzoites decreased under the EX527 treatment (Figure 2B). At the same time, SRT1720 promoted parasite survival, along with the rupture of cells and even the escape of tachyzoites (Figure 2B). Observation under TEM showed that the intracellular *T. gondii* gradually increased over time in comparison with the control (Figure 2C). There were more intracellular *T. gondii* in the SRT1720 group and fewer in the EX527 group compared to that in the control group (Figure 2C).

### The association of CD154/IFN-γ with SIRT1 on the killing of intracellular tachyzoites

**Figure 3:**
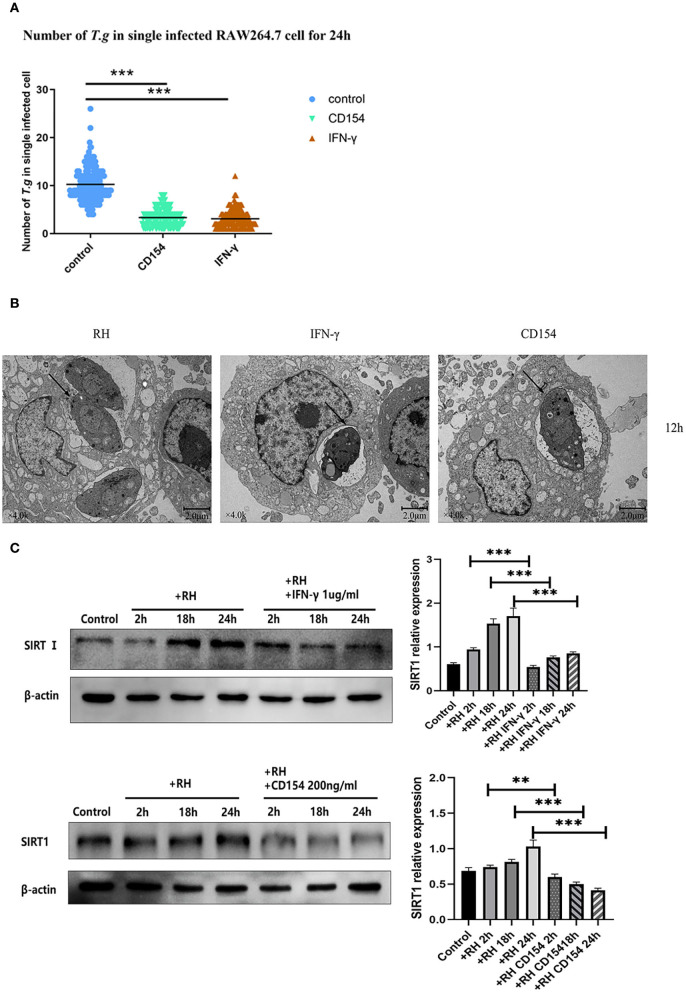
Intracellular growth of RH tachyzoites inside mouse macrophages with interferon gamma (IFN-γ) or CD154 *in vitro* and Sirtuin 1 (SIRT1) regulation-infected macrophages stimulated with IFN-γ and CD154 at different time points. RH tachyzoites were harvested to infect RAW 264.7 mouse macrophages at an infection ratio of 5:1 (parasite/host cell) for 2 h, followed by the removal of excess parasites and incubation with IFN-γ (1 μg/ml) or CD154 (200 ng/ml) for an extra 24 or 12 h before observation under light microscopy or transmission electron microscopy (TEM). RAW 264.7 macrophages were either infected with RH tachyzoites at a ratio of 5:1 (parasite/host cell) for 2 h or left in the culture plate without infection, followed by incubation with IFN-γ (1 μg/ml) or CD154 (200 ng/ml) for 2, 18, or 24 h before collection of the cell lysates. We adjusted the protein concentration of each group of samples to be consistent and then carried out the experiment. Anti-SIRT1 antibody and anti-beta-Actin antibody were used at a 1:1,000 dilution. **(A)** A scattered distribution chart showing the mean intracellular tachyzoite counts. Values represent actual numbers (scattered dots) and means (black line) from three independent experiments; in each case, at least 200 infected cells were counted. The interference of IFN-γ or CD154 reduced the average number of intracellular tachyzoites from 10 to about 3 at 24 h after the infection of *Toxoplasma gondii*. **(B)** Observation under TEM showed that the infected macrophages had much heavier intracellular tachyzoite burdens than infected macrophages incubated with IFN-γ and CD154 at 12 h after infection. The arrows indicate the tachyzoites inside host cells. **(C)** Infected macrophages treated with IFN-γ and CD154 showed a downregulation of SIRT1 2, 18, and 24 h compared to the *T. gondii* infection group. Among them, the expression level of SIRT1 exhibited its greatest decrement at 24 h. These results were obtained from three independent experiments. RH, *T. gondii* RH strain. **P < 0.01 and ***P < 0.001.

In humans and mice, IFN-γ played an important role in the formation of the parasitophorous vacuole (Choi et al., 2014; Park et al., 2016). At the same time, CD40, a member of the tumor necrosis factor (TNF) receptor superfamily, was also believed to induce the autophagic clearance of *T. gondii* by host cells, and this effect was independent of IFN-γ (Van Grol et al., 2013; Liu et al., 2016). To clarify whether SIRT1 might be associated with the clearance effects of CD40 or IFN-γ through the two classic killing pathways, we performed experiments using CD40L (CD154) or IFN-γ to observe the impact on parasite burden and also the alteration of SIRT1 expression. We confirmed in our model that both CD154 and IFN-γ were able to greatly reduce the intracellular RH tachyzoite burden in host cells (Figure 3A). The results of TEM further confirmed this conclusion (Figure 3B). Since SIRT1 interference and IFN-γ and CD154 stimulation affected the number of intracellular *T. gondii*, we explored whether IFN-γ and CD154 stimulation could affect SIRT1 in the case of *T. gondii* infection. We found that both IFN-γ and CD154 reduced the expression of SIRT1 during *T. gondii* infection of macrophages (Figure 3C).

**Figure 4:**
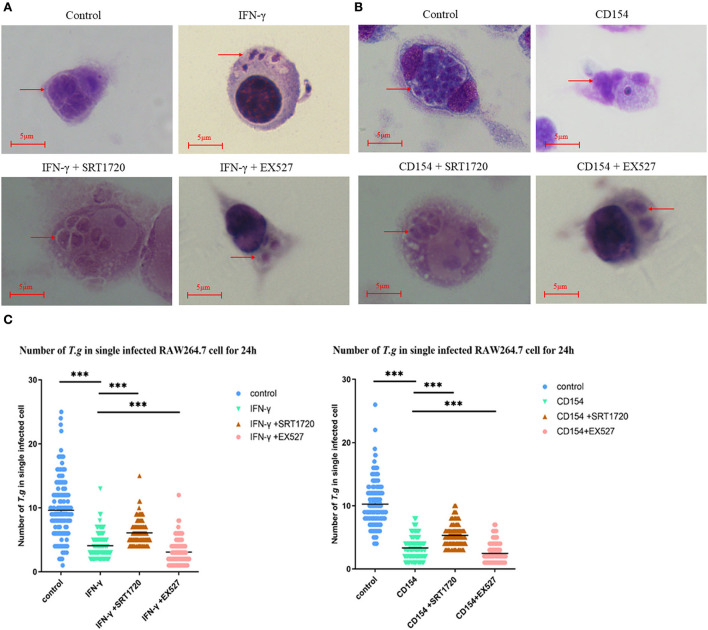
Intracellular growth of RH tachyzoites inside mouse macrophages with regulation by Sirtuin 1 (SIRT1) and interferon gamma (IFN-γ) or CD154 at different time points. RAW 264.7 macrophages were either infected with RH tachyzoites at a ratio of 5:1 (parasite/host cell) for 2 h or left in the culture plate without infection, followed by incubation with IFN-γ (1 μg/ml), CD154 (200 ng/ml), IFN-γ (1 μg/ml) and SRT1720 (5 μM), IFN-γ (1 μg/ml) and EX527 (10 μM), CD154 (200 ng/ml) and SRT1720 (5 μM), or CD154 (200 ng/ml) and EX527 (10 μM) for 24 h. Before observation under light microscopy. **(A)** Observation under light microscopy pointed out that the infected macrophages had much heavier intracellular tachyzoite burdens than infected macrophages incubated with IFN-γ at 24 h after infection. SRT1720 neutralized the intracellular tachyzoites' downward trend caused by IFN-γ, while EX527 enhanced the downtrend. **(B)** Observation under a light microscope revealed that the infected macrophages had much heavier intracellular tachyzoite burdens than infected macrophages incubated with CD154 at 24 h after infection. SRT1720 neutralized the intracellular tachyzoites' downward trend caused by IFN-γ, while EX527 enhanced the downtrend. **(C)** A scattered distribution chart showing the means of the intracellular tachyzoite counts. Values represent actual numbers (scattered dots) and means (black line) from three independent experiments; in each case, at least 200 infected cells were counted. ***P < 0.001.

The mean number of *T. gondii* tachyzoites was significantly reduced in IFN-γ-stimulated macrophages compared with that of the control group, with the number of tachyzoites in macrophages dropping from 9 to about 4 (Figure 4A). We further examined the combination of IFN-γ stimulations and SIRT1 interference in intracellular tachyzoites. The average number of tachyzoites in macrophages stimulated by IFN-γ plus EX527 had dropped approximately from 4 to 3, indicating that EX527 promoted the elimination effect initiated by IFN-γ (Figure 4C). Similarly, CD154 stimulation plus EX527 showed the same pattern as what was observed in IFN-γ stimulation (Figure 4B). In addition, the average number of tachyzoites in macrophages stimulated by IFN-γ plus SRT1720 increased approximately from 4 to 6, revealing that SRT1720 reverted the effect of IFN-γ (Figure 4C).

### IRGM1 regulation by IFN-γ and CD154 stimulations and the effect of SIRT1 on it

**Figure 5:**
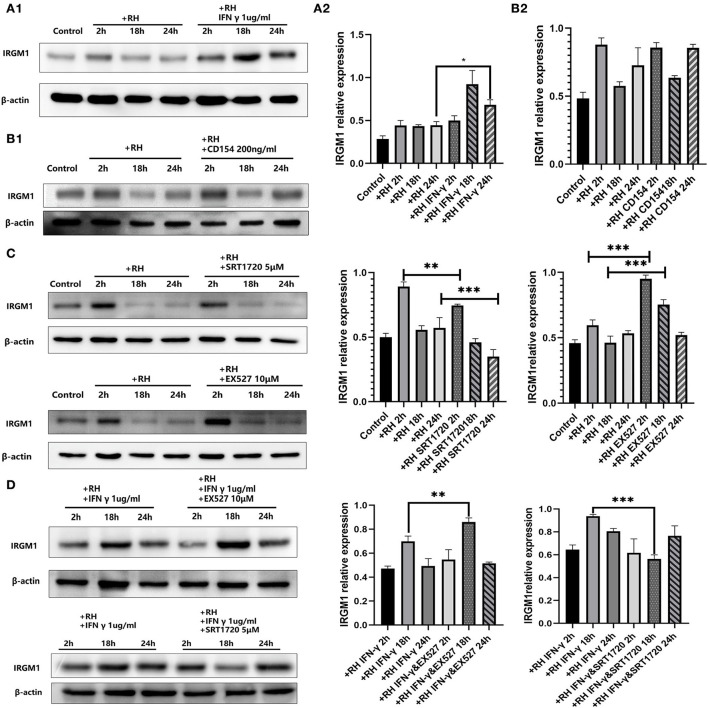
Murine ortholog of immunity-related GTPase family M (IRGM1) regulation by Sirtuin 1 (SIRT1) with or without interferon gamma (IFN-γ) or CD154 at different time points. RAW 264.7 macrophages were either infected with RH tachyzoites at a ratio of 5:1 (parasite/host cell) for 2 h or left in the culture plate without infection, followed by incubation with IFN-γ (1 μg/ml), CD154 (200 ng/ml), SRT1720 (5 μM), EX527 (10 μM), IFN-γ (1 μg/ml) and SRT1720 (5 μM), or IFN-γ (1 μg/ml) and EX527 (10 μM) for 2, 18, or 24 h before the collection of the cell lysates. We adjusted the protein concentration of each group of samples to be consistent and then carried out the experiment. Anti-IRGM1 antibody and anti-beta-Actin antibody were used at a 1:1,000 dilution. **(A)** The expression of IRGM1 in the *Toxoplasma gondii* infection group increased at 2 h but decreased at 18 and 24 h compared to that for the control group. Infected macrophages treated with IFN-γ displayed an upregulation of IRGM1 at 2, 18, and 24 h compared to that of the infection-only group. **(B)** Infected macrophages treated with CD154 showed no significant changes of IRGM1 compared to that for the infection-only group. **(C)** Infected macrophages treated with RH tachyzoites showed an upregulation of IRGM at 2 h and a downregulation at 18 or 24 h compared to that of the control group. Meanwhile, IRGM1 production was found to be decreased under SRT1720 stimulation and increased with the existence of EX527 at 2 h. However, both SRT1720 and EX527 failed to interfere with the IRGM1 level at 18 and 24 h. **(D)** When combining SIRT1 interference and IFN-γ stimulation, infected macrophages treated with IFN-γ and SRT720 showed a downregulation of IRGM at 2, 18, and 24 h compared to that of the IFN-γ group. Meanwhile, infected macrophages treated with IFN-γ and EX527 showed an upregulation of IRGM at 2, 18, and 24 h compared to that of the IFN-γ group. These results were obtained from three independent experiments. RH, *T. gondii* RH strain. *P < 0.05, **P < 0.01, and ***P < 0.001.

IRGM was proven to be one of the parasitophorous vacuole membrane cleavage proteins induced by IFN-γ, which led to the rupture of parasitophorous vacuoles and the degradation of *T. gondii* in the host cell (Chauhan et al., 2015). Meanwhile, CD40 was believed to induce autophagic clearance of *T. gondii* by host cells (Andrade et al., 2006). IRGM1 is the ortholog in murine cells of IRGM. In our model, IFN-γ was able to elevate the expression of IRGM1 (Figure 5A) while CD154 (the ligand of CD40, also known as CD40L) was not (Figure 5B).

We further examined whether IRGM1 had an association with SIRT1 with or without IFN-γ. We found that stimulating with SIRT1 slightly neutralized the upregulated IRGM1 level of the host cells, while inhibiting with SIRT1 appeared to have a slight increase in IRGM1 expression (Figure 5C). When we combined IFN-γ stimulation with SIRT1 interference, we observed that, only at 18 h but not at 24 h, stimulating SIRT1 with SRT1720 decreased the IRGM1 level, while inhibiting SIRT1 with EX527 increased IRGM1 (Figure 5D). Therefore, the results above indicated that SIRT1 was likely to regulate the killing of intracellular *T. gondii* by direct or indirect interaction with IRGM1 at certain time points.

### Autophagy regulation by SIRT1 interference and IFN-γ/CD154 stimulation

Autophagy is a conservative lysosomal degradation mechanism and serves as an important way for innate immunity to resist intracellular pathogens (Glick et al., 2010; Siqueira et al., 2018). Studies found that many common pathogenic bacteria and viruses might induce the autophagic clearance effects of host cells (Watson et al., 2012; Robinson et al., 2018). At the same time, pathogenic organisms were reported to inhibit or utilize autophagy in different ways to meet their survival and proliferation needs (Shrivastava et al., 2016; Mohamud et al., 2018). Moreover, studies confirmed that autophagy could downregulate intracellular SIRT1 under certain circumstances (Xu et al., 2020). However, many researchers pointed out that SIRT1 in cells activated autophagy through different mechanisms (Luo et al., 2019; Xu et al., 2020).

**Figure 6:**
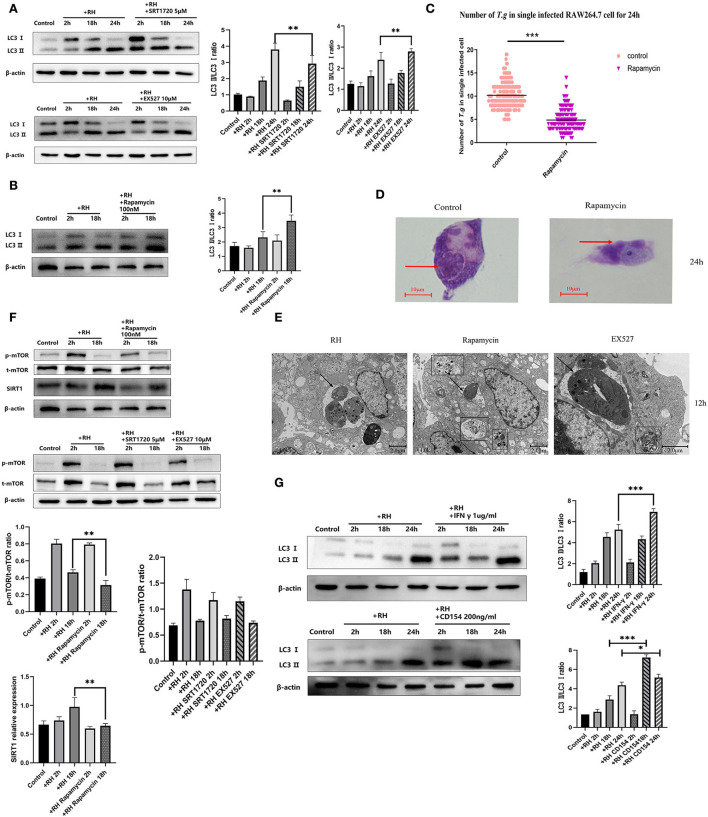
Autophagy regulation by Sirtuin 1 (SIRT1) and interferon gamma (IFN-γ) or CD154 at different time points. RAW 264.7 macrophages were either infected with RH tachyzoites at a ratio of 5:1 (parasite/host cell) for 2 h or left in the culture plate without infection, followed by incubation with SRT1720 (5 μM), EX527 (10 μM), or rapamycin (100 nM) for 2, 18, or 24 h before collection of the cell lysates or followed by incubation with rapamycin (100 nM) for 24 h for light microscopy or 12 h before transmission electron microscopy (TEM). We adjusted the protein concentration of each group of samples to be consistent and then carried out the experiment. Anti-LC3 I/LC3 II antibody, anti-phospho-mTOR (mechanistic target of rapamycin kinase) antibody, anti-mTOR, and anti-beta-Actin antibody were used at a 1:1,000 dilution. **(A)** The LC3 II/LC3 I ratio in the *Toxoplasma gondii* infection group increased over time compared to that for the negative control. Infected macrophages treated with SRT1720 showed a downregulation of LC3II/LC3 I at 2, 18, and 24 h compared to that for the *T. gondii* infection group. Infected macrophages treated with EX527 showed an upregulation of LC3II/LC3 I at 2, 18, and 24 h compared to that for the *T. gondii* infection group. **(B)** Infected macrophages treated with rapamycin showed an upregulation of LC3 II/LC3 I ratio at 2 and 18 h compared to that for the *T. gondii* infection group. **(C)** A scattered distribution chart showing the means of the intracellular tachyzoite counts. Values represent actual numbers (scattered dots) and means (black line) from two independent experiments; in each case, at least 200 infected cells were counted. **(D)** Observation under the light microscope showed that the infected macrophages had much heavier intracellular tachyzoite burdens than infected macrophages incubated with rapamycin at 24 h after infection. **(E)** Observation under TEM revealed that the infected macrophages had much heavier intracellular tachyzoite burdens than infected macrophages incubated with rapamycin at 12 h after infection. At the same time, host cells showed a large number of autophagosomes after being incubated with rapamycin for 12 h. These results were obtained from three independent experiments. Autophagosomes are multilayered structures containing cytoplasmic organelles. The arrows indicate the tachyzoites inside or outside host cells. The box indicates the autophagosome inside host cells. The values shown are the means ± SEM from three independent experiments. The arrows indicate the tachyzoites inside host cells. The box indicates the autophagosome inside host cells. **(F)** When infected by RH tachyzoites at two different time points, the mouse macrophages displayed an increased phosphorylation level of mTOR at 2 h and a decrease at 18 h. Infected macrophages treated with rapamycin showed a decrease in the mechanistic target of rapamycin kinase (mTOR) phosphorylation level at 2 and 18 h. At the same time, infected macrophages treated with rapamycin showed a downregulation of SIRT1 at 2 and 18 h compared to that of the infection-only group. Infected macrophages treated with SRT1720 or EX527 showed no significant changes of mTOR or phosphorylation level of mTOR compared to that of the infection-only group. p-mTOR, phosphorylated mTOR. t-mTOR, total mTOR. **(G)** The LC3 II/LC3 I ratio in the *T. gondii* infection group increased over time compared to that of the control group. Infected macrophages treated with IFN-γ showed an upregulation of LC3II/LC3 I at 2, 18, and 24 h compared to that for the *T. gondii* infection group. Infected macrophages treated with CD154 showed an upregulation of LC3II/LC3 I at 2 h and 18 h, but not at 24 h compared to the *T. gondii* infection group. These results were obtained from three independent experiments. RH, *T. gondii* RH strain. *P < 0.05, **P < 0.01, and ***P < 0.001.

We first observed whether infection with the RH strain altered the LC3 II/LC3 I ratio in host cells and found that the autophagic process of host cells was initiated by infection of RH tachyzoites over time (Figure 6A), leading to the appearance of autophagosomes and reduced number of tachyzoites (Figures 6D,E). After that, we analyzed the changes in host cell autophagy in cases of *T. gondii* infection by assessing the LC3 II/LC3 I ratio in the presence of SRT1720 or EX527. When SRT1720 was added, the host cell LC3 II/LC3 I ratio was downregulated. In contrast, when EX527 was added, the LC3 II/LC3 I ratio was upregulated, compared with that of the *T. gondii* infection-only group (Figure 6A). Furthermore, the EX527 group exhibited autophagosomes, confirming that the inhibition of SIRT1 promotes autophagy in host cells. These results together suggested that SIRT1 appeared to be inhibiting the autophagy process in infected macrophages.

As one of the important proteins in the autophagy signaling pathway, mTOR plays an important role in the regulation of autophagy clearance of host cells. Inhibition of mTOR has been reported to be one of the pathways that directly trigger autophagy (Kim and Guan, 2015). We wondered whether the effect of SIRT1 on autophagy was upstream or downstream of mTOR in *T. gondii* infection. Next, we used rapamycin (among various pathways known to regulate autophagy in mammalian cells, the best understood is the one regulated by mTOR (mechanistic target of rapamycin kinase), induction of autophagy can be achieved by the treatment of rapamycin) to stimulate *T. gondii*-infected host cells and found that rapamycin stimulation further upregulated the LC3 II/LC3 I ratio of host cells (Figure 6B). By counting the number of tachyzoites in each infected cell, we demonstrated that autophagy-activated macrophages had a greater ability to kill the intracellular parasites. After 24 h of infection, the activation of autophagy reduced the average number of intracellular *T. gondii* to a significantly low level (Figure 6C). mTOR inhibited with rapamycin showed that the expression level of SIRT1 decreased, while the interference of SIRT1 did not affect mTOR levels (Figure 6F), indicating that mTOR might intervene upstream of SIRT1 in regulating intracellular killing *T. gondii* by host macrophages. IFN-γ and CD154 have been reported to increase autophagy to mediate the host's resistance to pathogens (Wu et al., 2020). In this study, IFN-γ stimulation led to an increment in the LC3 II/LC3 I ratio of host cells after 24 h of infection, while CD154 stimulation promoted autophagy after 18 and 24 h of infection, compared with the control group (Figure 6G), indicating that IFN-γ and CD154 were able to promote autophagy at certain time points, while inhibition of SIRT1 had a supplementary role in these two processes.

## Discussion

*T. gondii* is an opportunistic protozoan that causes severe clinical symptoms in immunocompromised individuals (Zhou et al., 2011). Previous studies revealed that murine macrophages infected by *T. gondii* showed dynamic alterations in the levels of histone deacetylase SIRT1 with an increase in the duration of infection. At the same time, the introduction of resveratrol (a known natural product that regulated SIRT1) affected the number of intracellular tachyzoites (Chen et al., 2019). Based on these previous findings, we proposed that SIRT1 might play a fundamental role in the elimination of host cell toward *T. gondii* and also in the invasion and immune evasion of the parasites toward the host. Therefore, we tried to clarify the role and the mechanism of SIRT1 in *T. gondii* infection of host macrophages to assess the potential of SIRT1 as a promising therapeutic target for clinical application.

SIRT1 is the first discovered molecule in the mammalian sirtuin family (Chen et al., 2015). Different parasites have been reported to affect SIRT1 or be affected by SIRT1 through various mechanisms so as to evade from the host's immune response. In our experiments, SIRT1 expression and activity (regulated by the NAD^+^/NADH ratio) was increased by *T. gondii* infection, and if SIRT1 was stimulated pharmacologically, *T. gondii* increased in number, but the opposite effect was demonstrated by the inhibition of SIRT1. This result is in contrast to our previous study, which showed that resveratrol, which activates SIRT1, caused a decrease in the number of intracellular *T. gondii* tachyzoites (Chen et al., 2019). We believe that the reason for the contradiction is that resveratrol does not only act on SIRT1 (Beher et al., 2009), and the mechanism of its inhibition of intracellular *T. gondii* is not by activating SIRT1. The results of confocal microscopy implied that the SIRT1 expression in RAW 264.7 cells had a tendency to translocate from the nucleus to the cytoplasm, suggesting that *T.gondii* infection affected the localization of SIRT1 in host cells, which might be caused by the killing effect of host cells toward intracellular tachyzoites or by the immune evasion of *T. gondii* (Xu et al., 2020). Considering that the activation and aggregation of lysosomes might also be altered by the killing effect of macrophages on intracellular tachyzoites, we conducted an interference experiment on the expression level of SIRT1 in *T. gondii*-infected macrophages. Our results showed that the inhibition of SIRT1 by EX527 in host cells significantly reduced the average number of intracellular tachyzoites to a significantly low level. On the contrary, when SRT1720 was used to stimulate SIRT1 in host cells, the proliferation of intracellular tachyzoites was significantly increased, leading to the unsuccessful counting of intracellular tachyzoites due to cellular rupture. Shalini Roy and colleagues observed that the late phase of *Leishmania* infection also induced SIRT1 expression and found that SIRT1-mediated deacetylation of Forkhead box protein 1 (FOXO-1) subsequently prevented apoptosis (Roy et al., 2019). Xianxiu Wan and colleagues found that pro-inflammatory macrophages were reprogrammed to a healing phenotype by enhancing the SIRT1 activity and by inhibiting focal adhesion kinase (FAK) signaling in macrophage proliferation and activation of a pro-inflammatory reaction in Chagas disease caused by *Trypanosoma cruzi* (Wan et al., 2019). Several studies showed that SIRT1 was mainly distributed in the nucleus, but recent articles argued that SIRT1 was likely to distribute elsewhere in the cytoplasm and play several physiological roles (Xu et al., 2020). Similarly, Moreira D found that the stimulation of SIRT1 increased *L. infantum* infection rates when infecting bone marrow mononuclear (BMMo) cells, whereas Sirtuin 1 knockout bone marrow mononuclear (SIRT1 KO BMMo) cells exhibited lower *L. infantum* infection rates (Moreira et al., 2015). These results indicated that, in *T. gondii* infection, host cell SIRT1 might be utilized by *T. gondii* to achieve proliferation and immune evasion. Inhibition of SIRT1 in host cells can mediate the clearance of intracellular *T. gondii* through a certain pathway.

We stimulated murine macrophages infected by *T. gondii* with IFN-γ and CD154 (CD40 ligand) and confirmed that both lead to a significant reduction in the number of intracellular *T. gondii*. Next, we found out that the initiation of these two classic parasite killing pathways led to the inhibition of SIRT1. This suggested that the inhibition of SIRT1 might be involved in the IFN-γ-dependent/CD40-dependent intracellular clearance of *T. gondii*. IFN-γ-stimulated cells expressed hundreds of IFN-stimulated-related genes, including members of the guanosine triphosphatase (GTPase) family. Immune-related GTPases (IRGs) belong to the IFN-γ-inducible GTPase family and play important roles in IFN-γ-mediated host anti-infection and inflammatory responses (Taylor, 2007). Recruitment of IRGs (immune-related GTPases) to the pathogen-containing vacuole (PV) membrane led to their destruction and subsequent destruction of the parasite on exposure to the host's cytoplasm (Howard et al., 2011). Li et al. (2012) found that IFN-γ can inhibit the transcription of SIRT1, which can further affect the energy metabolism homeostasis of cells. Moreover, we observed that SIRT1 was downregulated by IFN-γ, and irrespective of the presence of IFN-γ, activating SIRT1 inhibited the expression of IRGM1, and inhibiting SIRT1 increased the expression of IRGM1. These results indicated that SIRT1 played a role in the IRGM1-induced clearance process triggered by IFN-γ or through other pathways with SIRT1 as a link. Interestingly, the change of IRGM1 mainly occurred at 18 h rather than 24 h. We believe that the strong virulence of the RH strain *T. gondii* leads to a decrease in the IRGM1 level of host cells at 24 h. Therefore, the change of IRGM1 at 24 h was not obvious. However, CD154 seemed to have no interaction with IRGM1, but it was observed to downregulate SIRT1, which indicated that CD154 and CD40 eliminated the parasites through other pathways. In fact, a CD40-dependent autophagy mechanism that effectively killed intracellular *T. gondii* has been reported (Andrade et al., 2006). Pan et al. (2016) found that the expression of SIRT1 suppressed CD40 expression in human umbilical endothelial cells (HUVECs), whereas the suppression of SIRT1 can reduce this inhibitory effect. Altogether, the inhibition of SIRT1 was assumed to participate in the IFN-γ-dependent/CD40-dependent clearance of intracellular *T. gondii*. Inhibition of SIRT1 might assist in IFN-γ-dependent clearance of intracellular *T. gondii* by upregulating IRGM1.

Although the clearance mechanism of CD40 was different from the traditional IFN-γ-mediated mechanism, it also required the assistance of host cell autophagy-related proteins to force the PV to bind to lysosomes, thereby completing the degradation work (Andrade et al., 2006). We directly stimulated mouse macrophages with rapamycin to induce autophagy, and the results revealed that rapamycin initiated autophagy in host cells and had a significant clearance effect on intracellular *T. gondii*. This confirmed that autophagy-related clearance was essential for the elimination of intracellular *T. gondii*. Next, we examined the influence of SIRT1 interference on host cell autophagy. The results showed that inhibiting SIRT1 promoted autophagy while stimulating SIRT1 inhibited autophagy, indicating that the interference of SIRT1 affected the clearance of intracellular *T. gondii* through the regulation of autophagy. Existing studies on the effect of SIRT1 in infection by pathogenic microorganisms and host autophagic clearance showed that *Salmonella typhimurium* induced SIRT1 to translocate from the nucleus to the cytoplasm and destroyed its indirect activation of AMP-activated kinase (AMPK) after infecting macrophages, thereby inhibiting autophagy in host cells (Ganesan et al., 2017). In a macrophage model of *Leishmania infantum* infection, it was found that intracellular SIRT1 regulated the activation of AMPK, and the deletion of intracellular SIRT1 and AMPK in the middle and late infection stages contributes to the clearance of parasites (Moreira et al., 2015). However, Lee et al. (2020) reported that 4-hydroxybutyl acrylate (4-HBA) promoted antiparasitic host responses by activating SIRT1-mediated autophagy, which was in contrast to our experimental results. We attributed this conflict to the difference in the proportion and time points of *T. gondii*, and the authors neglected the changes in SIRT1 under *T. gondii* infection, while in our study it was found that *T. gondii* infection caused SIRT1 changes in host cells, thereby further affecting the immune response of host cells. In addition, we measured the inhibition rate of *T. gondii* by directly counting the number of tachyzoites in the cells, while Lee et al. (2020) measured the co-localization of LC3 with *T. gondii*. After using rapamycin to inhibit mTOR in host cells infected by *T. gondii*, we found that SIRT1 was downregulated along with mTOR inhibition. However, our study did not lead to any significant changes in mTOR following SIRT1 modulation. This finding indicated that SIRT1 was located downstream of mTOR during the clearance of intracellular *T. gondii* caused by autophagy. Then, we verified whether the clearance of *T. gondii* by IFN-γ and CD154 was effected through the autophagy pathway, and the results demonstrated that CD154 enhanced *T. gondii* clearance by boosting the autophagy process at some stage, while IFN-γ eliminated *T. gondii* mainly through IRGM1 and by partially initiating the autophagy process. Collectively, the inhibition of SIRT1 may play a role in IFN-γ and CD154-mediated autophagy against the *T. gondii* infection in RAW 264.7 macrophages.

**Figure 7:**
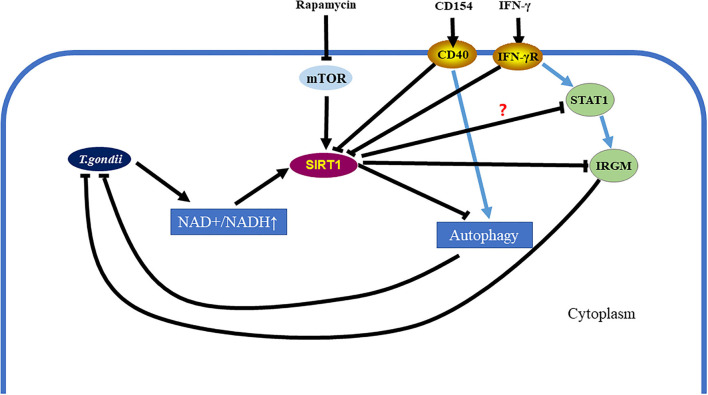
*Toxoplasma gondii* can be eliminated in infected cells by inhibiting the Sirtuin1 (SIRT1) protein. After *T. gondii* invades host cells, it causes the upregulation of the nicotinamide adenine dinucleotide (NAD^+^/NADH) ratio in host cells, which further leads to the upregulation of SIRT1. However, SIRT1 inhibits parasitic clearance *via* the immunity-related GTPase family M (IRGM) pathway and the activation of autophagy. Interferon gamma (IFN-γ) stimulation can clear intracellular *T. gondii* by simultaneously upregulating IRGM and activating autophagy. CD154 stimulation can clear intracellular *T. gondii* by activating autophagy. These two methods of eliminating intracellular *T. gondii* are assisted by inhibiting SIRT1. Directly using rapamycin to inhibit the mechanistic target of rapamycin kinase (mTOR) and initiate autophagy is also a way to eliminate intracellular *T. gondii*, which is also accompanied by SIRT1 inhibition.

In conclusion, our study demonstrates that SIRT1 contributes to the complex process of *T. gondii* invasion of host cells and immune response to *T. gondii*. Moreover, *T. gondii* mediates SIRT1 for the purpose of survival and multiplication. Activation of SIRT1 leads to the proliferation of parasites, while the inhibition of SIRT1 promotes parasite elimination by host cells. During the invasion of host cells by *T. gondii*, SIRT1 may affect the clearance of *T. gondii* by the following ways: SIRT1 inhibits the clearance of intracellular *T. gondii* by inhibiting IRGM1, and/or SIRT1 hampers the killing of intracellular *T. gondii* by downregulating the activation of autophagy, which is one of the important pathways responsible for the elimination of intracellular parasites (Figure 7). At the same time, recent studies also reported that a potent HDAC inhibitor can also inhibit the growth of *T. gondii in vivo* (Mouveaux et al., 2022), which may also provide some ideas for further exploring the mechanism of inhibiting intracellular *T. gondii* tachyzoites by inhibiting SIRT1. In addition, IFN-γ can upregulate IRG by mediating signal transducer and activator of transcription 1 (STAT1) to resist *T. gondii* infection (Hakimi, 2022), and this also provides another possibility to explore the mechanism of inhibition of SIRT1 in resistance to *T. gondii* infection (Figure 7).

## Data availability statement

The original contributions presented in the study are included in the article/supplementary material, further inquiries can be directed to the corresponding author/s.

## Author contributions

All authors listed have made a substantial, direct, and intellectual contribution to the work and approved it for publication.

## Funding

This work was supported by the National Natural Science Foundation of China (Grant Nos. 81902086 and 81672048).

## Conflict of interest

The authors declare that the research was conducted in the absence of any commercial or financial relationships that could be construed as a potential conflict of interest.

## Publisher's note

All claims expressed in this article are solely those of the authors and do not necessarily represent those of their affiliated organizations, or those of the publisher, the editors and the reviewers. Any product that may be evaluated in this article, or claim that may be made by its manufacturer, is not guaranteed or endorsed by the publisher.
